# In vivo real-time positron emission particle tracking (PEPT) and single particle PET

**DOI:** 10.1038/s41565-023-01589-8

**Published:** 2024-01-19

**Authors:** Juan Pellico, Laurence Vass, Amaia Carrascal-Miniño, Francis Man, Jana Kim, Kavitha Sunassee, David Parker, Philip J. Blower, Paul K. Marsden, Rafael T. M. de Rosales

**Affiliations:** 1 School of Biomedical Engineering and Imaging Sciences, King’s College London, London, UK; 2 School of Physics and Astronomy, University of Birmingham, Birmingham, UK

**Keywords:** Nanoparticles, Biomedical engineering

## Abstract

Positron emission particle tracking (PEPT) enables 3D localization and tracking of single positron-emitting radiolabelled particles with high spatiotemporal resolution. The translation of PEPT to the biomedical imaging field has been limited due to the lack of methods to radiolabel biocompatible particles with sufficient specific activity and protocols to isolate a single particle in the sub-micrometre size range, below the threshold for capillary embolization. Here we report two key developments: the synthesis and ^68^Ga-radiolabelling of homogeneous silica particles of 950 nm diameter with unprecedented specific activities (2.1 ± 1.4 kBq per particle), and the isolation and manipulation of a single particle. We have combined these developments to perform in vivo PEPT and dynamic positron emission tomography (PET) imaging of a single radiolabelled sub-micrometre size particle using a pre-clinical positron emission tomography/computed tomography scanner. This work opens possibilities for quantitative assessment of haemodynamics in vivo in real time, at the whole-body level using minimal amounts of injected radioactive dose and material.

## Abstract (web summary)

In vivo positron emission particle tracking (PEPT) remains a challenge due to the lack of single-particle tracers. Here a sub-micrometre silica particle has been radiolabelled and isolated with high specific activity, allowing the dynamic tracing of a single particle in vivo using PEPT in mice.

## Main

Positron emission particle tracking (PEPT) allows determination of the three-dimensional location and trajectory of a moving single particle with high spatiotemporal resolution within multiphase, large, dense and/or optically opaque systems^1^. This is fundamentally different to positron emission tomography (PET). In PET, the γ-rays generated by positron annihilation are registered to provide an image of the radiotracer (radiopharmaceutical) distribution within the body as an average of the location of many molecules/particles. PEPT, however, uses those γ-rays for the fast accurate localization of a single radiolabelled particle taking advantage of the knowledge that all the emitted γ-rays originate from the same point source.

Currently, PEPT is restricted to industrial applications such as the evaluation of particle–fluid or particle–particle interactions in the pharmaceutical, chemical, oil, mining, construction and power generation industries, among others^2–4^. Due to the extraordinary spatiotemporal resolution of PEPT, the movement of the particle can be tracked at high velocities, and in a quantitative manner. The velocity at which a particle can be tracked by PEPT is largely dependent on the amount of radioactivity per particle, and the sensitivity of the PEPT camera. With the right combination, the results achievable are impressive; for example, the Forte camera at the University of Birmingham (United Kingdom) allows the tracking of a particle travelling at 1 m s^−1^ to within 0.5 mm approximately 250 times a second^5^. Recent developments in this area such as the superPEPT camera are likely to provide improved spatiotemporal resolutions^6^.

The industrial applications of PEPT anticipate exciting biomedical applications in the study of the velocity, density and overall dynamics of blood flow that are currently impossible to study by any other imaging modality. In vivo PEPT has the potential to provide important breakthroughs in the evaluation of abnormal events in cardiovascular diseases or cancer where the blood flow has a prominent impact. Our ambition is to explore the potential of PEPT in biomedicine to provide whole-body information about blood flow dynamics in different settings, with unique applications such as the study of complex multiphase flow of blood, crucial in clinical physiology and drug delivery. Moreover, in vivo PEPT with single radiolabelled cells would open the possibility to evaluate in deep detail the motion and migration of individual cells, and their interaction with blood vessels and tissues^7^. This is in contrast to standard clinical imaging techniques, such as nuclear imaging or magnetic resonance imaging, that can only provide information of the average location of millions of cells and cannot track individual cell trajectories.

The limiting factor that has prevented progress in biomedical in vivo PEPT is the lack of methods to radiolabel and isolate a single particle of a suitable composition, size and radioactivity concentration (specific activity). So far, there is one reported related study tracking a single cancer cell using whole-body PET^7^. This work describes the radiolabelling of breast cancer cells with ^68^Ga-labelled mesoporous silica nanoparticles and isolation of a single cell. The approach provided radiolabelled single cells with specific activities of 30–110 Bq per cell that were imaged and tracked by PET/computed tomography (CT). The imaging revealed the uptake of the cell in the lungs, and most importantly, the tracking demonstrated a rapid uptake within 2–3 s of tail vein injection and an average cell velocity of 50 mm s^−^^1^, consistent with the blood flow rate. This work showcases the possibilities of PEPT in biomedicine. The use of living cells as PEPT tracers, however, imposes restrictions on the levels of radioactivity per cell to prevent radiation-induced cell damage and death. Importantly, in PEPT, the amount of radioactivity of the single particle is directly related to the number of lines of response (LoRs) generated, and to the certainty to track its true position over time^8^. Hence, the limited amount of radioactivity that cells can carry restricts their detectability at high speeds (for example, heart flow) and prevents real-time tracking. Thus, it is highly desirable to maximize the radioactivity per single carrier to fully exploit the potential of in vivo PEPT.

The limitations of single cells as PEPT tracers could be overcome by using a single inorganic particle in the sub-micrometre or nanometric size range (100–1,000 nm). Inorganic particles are much less susceptible to radiolysis than living cells, and therefore, albeit dependent on the material/radionuclide combination and the radiolabelling method, high concentrations of radioactivity per particle can be achieved. In addition, their physicochemical properties (size, shape, charge and surface properties) can be finely controlled allowing a better control over the pharmacokinetics and biodistribution. Nevertheless, several challenges must be overcome before being able to image and track a single particle in vivo. First, an adequate combination of material and radionuclide with optimal synthetic and radiolabelling approaches is required. Second, methods to isolate and manipulate a single sub-micrometre particle, and inject it into a subject, remain a substantial obstacle.

In this Article, we present our efforts to develop the first single radiolabelled particle for biomedical PEPT. We evaluated different material–radionuclide combinations applying chelator-based and non-chelator radiolabelling strategies^9^. These combinations were surveyed applying previously developed strategies^10–14^. This preliminary study identified the combination of ^68^Ga–silica as the best candidate owing to the superb affinity of ^68^Ga towards silica materials after a simple non-chelator radiolabelling reaction^15^. Once the material and the radionuclide were identified, we aimed to: (1) synthesize silica particles with adequate physicochemical properties; (2) optimize the radiolabelling method to boost the specific activity; (3) find a method to isolate and manipulate a single particle; and (4) develop an imaging reconstruction method to track the position of the particle in a dynamic manner, to finally combine these in harmony to produce an image of a moving blood-borne particle in vivo.

## Results

### Synthesis and radiolabelling of smSiP

**Fig. 1:**
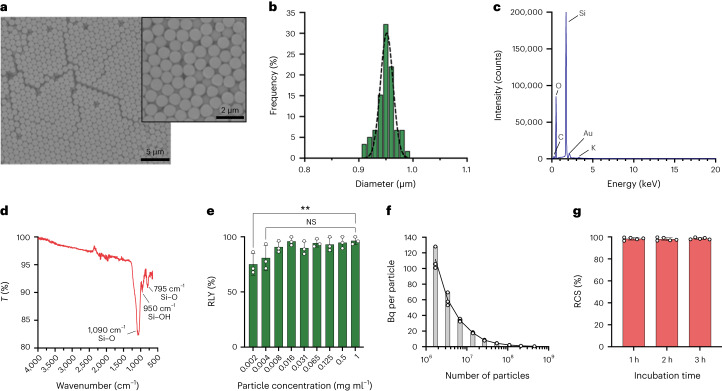
Physicochemical characterization and radiochemical properties. **a**, Representative SEM images of smSiP at two different magnification scales (left image scale bar, 5 µm; right image scale bar, 2 µm). **b**, Frequency distribution after analysis of 50 individual smSiP. **c**, EDS analysis of smSiP showing main peaks corresponding to Si and O and small peaks attributed to the grid components, C and Au. **d**, FT-IR transmission (*T*) spectrum of smSiP showing characteristic peaks for Si–O bonds at 1,090, 950 and 795 cm^−1^. **e**, RLY for ^68^Ga-smSiP at different particle concentrations, from 0.002 to 1 mg ml^−1^ (*n* = 3, non-significant (NS) *P* = 0.0542, **P = 0.0058, ordinary one-way ANOVA with Dunnett’s multiple comparisons test). **f**, Estimated average radioactivity per particle (Bq per particle) for the ^68^Ga-smSiP concentrations used for the radiolabelling, as a function of number of particles in the sample. **g**, RCS of ^68^Ga-smSiP at 2 µg ml^−1^ after incubation for 1, 2 and 3 h in human serum at 37 °C (*n* = 5). Data are represented as mean ± s.d. for **e** and **g**. Data represented as only mean for **f**. Source data

Sub-micrometre-size silica particles (smSiP) were synthesized following a modification of the Stöber method (details can be found in Methods). We obtained extremely homogeneous silica particles in the sub-micrometre size range as confirmed by scanning electron microscopy (SEM; Fig. 1a). The size of the particles was determined as 0.95 ± 0.05 µm by measuring the mean size of 50 particles (Fig. 1b). *ζ*-Potential measurements showed a surface charge of −41.1 ± 3.3 mV consistent with the presence of silanol groups on the surface (Supplementary Fig. 1). Energy-dispersive X-ray spectroscopy (EDS) revealed high-purity SiO_2_ with no other elements present in the sample other than those belonging to silica and the microscope grid (Fig. 1c). Fourier-transform infra-red spectroscopy (FT-IR) measurements confirmed the composition with a main absorption band at 1,090 cm^−1^ due to the asymmetric vibration of the Si–O bond, and bands at 950 cm^−1^ and 795 cm^−1^ that correspond to the asymmetric vibration of Si–OH and symmetric vibration of Si–O, respectively (Fig. 1d). Radiolabelling reactions with ^68^Ga were conducted to evaluate the affinity and binding capacity of smSiP. Particle suspensions at different mass/volume concentrations were incubated with [^68^Ga]GaCl_3_ eluted from a ^68^Ga/^68^Ga generator. To optimize the radiolabelling reaction, the generator eluate was first concentrated using an optimized cation exchange method (Supplementary Fig. 2a). The ^68^Ga eluate (4 ml) was passed through a cation exchange column at two different flow rates (2 and 1 ml min^−1^ leading to elution times of 5 and 10 min, respectively) controlled by a vacuum pump. A 10 min concentration step was sufficient to provide a recovery yield of 86.2 ± 8.5% (Supplementary Fig. 2b) in a final volume of 50 µl. The ^68^Ga solution was diluted in a small volume of the particle suspensions at different concentrations and the radiolabelling yield (RLY) measured by radio thin-layer chromatography (radio-TLC; Supplementary Fig. 3). RLY was 90–100% for typical particle concentrations of 0.125–1 mg ml^−1^ and 75.0 ± 9.2% at much lower particle concentrations (2 µg ml^−1^; Fig. 1e), with radiochemical purity (RCP; evaluated by radio-TLC after the purification of the particles) of 98.2 ± 1.6% (Supplementary Fig. 4). We then calculated the radioactivity per particle using a theoretical estimated number of particles and the radioactivity recovered after the radiolabelling reactions, calculating the average activity per particle. The number of particles was calculated considering a spherical shape and the density of silica (2.2 g cm^−^^3^) (Supplementary Scheme 1). The radioactivity per particle increases with decreasing particle concentration (Fig. 1f). Additionally, the radiochemical stability (RCS) was evaluated at 1, 2 and 3 h post-incubation in human serum at 37 °C and remained high at 98.5 ± 1.0% after 3 h (Fig. 1g).

### Particle quantification and radiolabelling optimization

Having optimized the preparation and established the suitability of the silica–^68^Ga combination, we used flow cytometry to precisely control the number of particles and isolate a single radiolabelled particle.

**Fig. 2:**
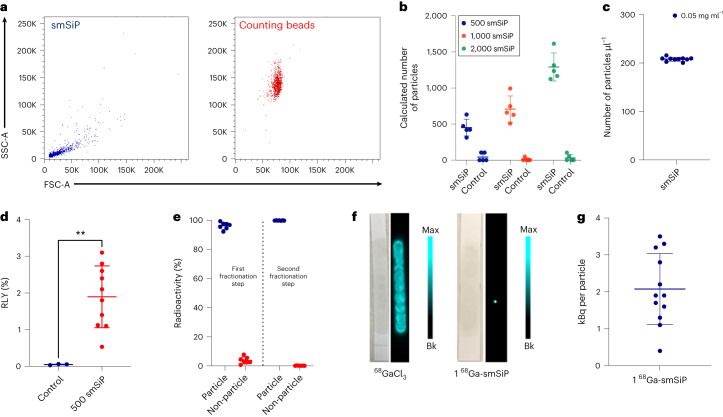
Quantification and radiolabelling of 500 smSiP. **a**, Side (SSC) versus forward (FSC) scatter plots showing the differences between smSiP and counting bead populations. **b**, Number of smSiP experimentally observed in smSiP–counting beads or PBS 1×–counting beads mixtures after the sorting of 500, 1,000 and 2,000 particles from smSiP at 0.05 mg ml^−1^ and PBS 1× solutions (*n* = 5). **c**, Number of smSiP per microlitre calculated from smSiP at 0.05 mg ml^−1^ and counting beads solutions (*n* = 10). **d**, RLY for the control reaction (reaction without particles, *n* = 3) and 500 ^68^Ga-smSiP (*n* = 10, *P* < 0.01, two-tailed Student’s *t*-test). **e**, Percentage of radioactivity in the ^68^Ga-smSiP particle and non-particle fractions measured by gamma counter after the first (left) and second (right) fractionation steps showing the difference in percentage of radioactivity between the single ^68^Ga-smSiP fraction and the other three fractions (*n* = 7). **f**, Picture of the solution spread along the TLC plate and the correspondent autoradiography image acquired after the addition of 1 ^68^Ga-smSiP suspended in 12.5 µl of PBS (right) and the addition of 12.5 µl of ^68^GaCl_3_ in PBS (left). Bk, background **g**, Specific radioactivity (kBq) for the isolated (single) ^68^Ga-smSiP (*n* = 12). Data are represented as mean ± s.d. Source data

Initially, we explored sorting samples containing 500, 1,000 and 2,000 particles from a starting smSiP suspension at 0.05 mg ml^−1^. Phosphate-buffered saline (PBS 1×) was used as a control to check for background light scattering. Counting beads (CountBright) were used in combination with smSiP samples (Fig. 2a) to provide precise quantification of the number of particles in suspension. The calculations revealed a larger deviation in the experimental number of particles when the number of particles sorted increased (Fig. 2b). When the instrument was set to sort 500 particles, an experimental number of particles of 452 ± 160 was obtained, while the particle numbers obtained after 1,000- and 2,000-particle sorts were 708 ± 243 and 1,291 ± 245, respectively. Additionally, control experiments did not show an appreciable number of particles, confirming that the counted particles are due to the presence of smSiP (Fig. 2b). These calculations suggest that using a cell sorter to isolate a specific number of smSiP could lead to large uncertainties in the real number of particles and might not be optimal for precise control over the number of particles. Therefore, we evaluated its ability to quantify the concentration of particles in specific suspensions. The same approach was followed to quantify the number of particles in ten independent solutions of smSiP at 0.05 mg ml^−1^. In this case, the counting method provided very consistent results with 208 ± 8 particles µl^−1^ (*n* = 10) (Fig. 2c).

We then chose to optimize the radiolabelling for 500 particles, aiming for a single particle with a potential radioactivity in the range of 100–1,000 Bq. Radio-TLC chromatograms showed low amounts of colloidal ^68^Ga after the elution of the ^68^Ge/^68^Ga generator but a prominent presence once the ^68^Ga elution is highly concentrated. Purification with decreasing concentrations of ethylenediaminetetraacetic acid (EDTA) successfully removed all unreacted and colloidal ^68^Ga providing highly pure radiolabelled particles (Supplementary Fig. 5) with an RLY of 1.9 ± 1.3% (Fig. 2d). Control reactions following the same procedure in the absence of the particles showed RLY <0.1% which confirms the 500 smSiP as the driving force of the radiolabelling (Fig. 2d). Then, a fractionation protocol was developed to isolate a single particle from the radiolabelling mixture. Different volumes of the mixture at a theoretical expected concentration of 1 particle µl^−1^ were added to different sample tubes and brought to the same final total volume, split into four equal parts and the radioactivity in each fraction measured in a gamma counter. A single particle was thus identified within the group of fractions in which most of the radioactivity was in one tube (particle fraction) with the other three only measuring residual radioactivity (non-particle fraction) (Fig. 2e, left). A second fractionation step was used to remove the residual activity found in the non-particle fractions. After this process, all the radioactivity was found in the particle fraction, confirming the purity of the sample (Fig. 2e, right). The fraction containing the radioactivity was spread along a TLC strip and imaged by autoradiography, revealing a single, small hotspot and confirming the presence of a single radiolabelled particle (Fig. 2f, right). This is in contrast to a control experiment with ^68^GaCl_3_, where the radioactivity smears along the strip (Fig. 2f, left). The specific activity calculated for the single particle was 2.1 ± 1.4 kBq per particle (Fig. 2g).

### In vitro and in vivo PET imaging

**Fig. 3:**
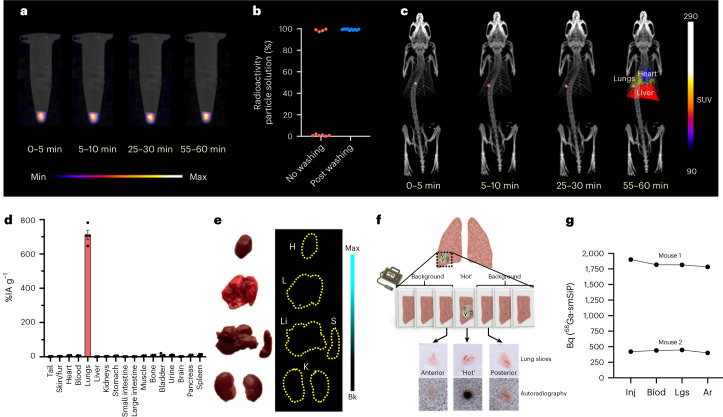
In vitro and in vivo imaging. **a**, In vitro PET/CT imaging 0–5, 5–10, 25–30 and 55–60 min of acquisition of a single 2.1 kBq ^68^Ga-smSiP suspended in 50 µl of PBS 1× in a sample tube showing enough sensitivity of the scanner to detect that level of radioactivity. **b**, Percentage of radioactivity in the particle solution after a single ^68^Ga-smSiP (suspended in 100 µl PBS) was individually passed through a cannula, without or with further washing with an additional 50 µl of PBS 1× (*n* = 10), showing that in six out of ten cases the particle successfully passed through the cannula and that in ten out of ten cases the particle passed through the cannula after a flushing step. Data represented as individual values. **c**, In vivo PET/CT imaging of a BALB/c mouse at 0–5, 5–10, 25–30 and 55–60 min after intravenous injection of a single 1.5 kBq ^68^Ga-smSiP. Regions of interests (ROIs) were drawn and represented in 55–60 min to delimit the lungs (yellow), the heart (blue) and the liver (red) for better clarity. Scale bar represents the standardised uptake value (SUV). **d**, Gamma counter biodistribution represented as %IA g^−1^ (injected activity per gram of tissue) 120 min after intravenous injection of a single 0.4–1.9 kBq ^68^Ga-smSiP (*n* = 4) showing radioactive signal only in the lungs. **e**, Autoradiography conducted for as-excised organs (H, heart; L, lungs; S, spleen; Li, liver; K, kidneys) with the radioactive signal represented in a blue scale, data represented as mean ± s.d. **f**, Autoradiography of 20 µm slices of the lung. The radioactivity was monitored for each slice, and the autoradiography was carried out on the ‘hot’ slice and the two slices adjacent to it. Radioactive signal, represented in black within the tissue slices, above background was only found in the ‘hot’ slice. **g**, Quantification of the radioactivity in Bq for the different steps involved during the in vivo experiments for mouse 1 injected with 1.9 kBq of ^68^Ga-smSiP and mouse 2 injected with 0.42 kBq of ^68^Ga-smSiP (*n* = 2). All the measurements are decay-corrected to the injection time for better comparison. Inj, activity measured in a gamma counter before the injection; Biod, activity in the lungs measured by gamma counter during the biodistribution studies; Lgs, activity in the 20 µm lung tissue slice measured by gamma counter; Ar, activity in the 20 µm lung tissue slice measured by autoradiography. Source data

To test whether the sensitivity of the pre-clinical PET scanner was sufficient to provide high-quality images with this level of radioactivity, a sample tube with a single 2.1 kBq ^68^Ga-smSiP was imaged for 2 h. The images demonstrated sufficient sensitivity at different 5-min time frames (Fig. 3a). Before the in vivo imaging, the behaviour of the single particle when injected through a cannula was evaluated. This was considered important since it is possible for the particle to be lost by adhesion to labware during sample preparation and injection. A single ^68^Ga-smSiP suspended in 100 µl of PBS was passed through a cannula and collected in a sample tube. After measuring the radioactivity in the collected solution and in the cannula, the particle was detected in the cannula six out of ten times. Consistent recovery (100%) of the particle from the cannula outlet was achieved by washing with an additional 50 µl of PBS after the injection (Fig. 3b). PET/CT imaging was then carried out in healthy BALB/c mice. Dynamic PET reconstructions of a mouse injected with a 1.5 kBq ^68^Ga-smSiP clearly revealed a single hotspot in the lung at only 5 min post-injection. Interestingly, this particle experienced a subtle movement to an inferior right part of the lungs between 5 min and 10 min post-injection, remaining static afterwards (Fig. 3c). In three replicate experiments, mice were injected with 0.4–1.9 kBq of ^68^Ga-smSiP showing similar imaging results, with the particle in a static position within the lungs (Supplementary Fig. 6). For each time frame of the PET reconstructions, a region of interest was outlined around the particle to quantify the signal. The decay corrected mean value of activity was 97 ± 3% of the injected activity. While the activity quantification in PET may not seem relevant for the end application of in vivo PEPT, it does show that the signal could be well characterized at low activities (Supplementary Fig. 7). Further biodistribution studies corroborated radioactive uptake exclusively in the lungs (Fig. 3d). Then, the heart, lungs, liver, spleen and kidneys were imaged together by phosphor imaging and showed a single, small spot signal in the inferior right lobe of the lung (Fig. 3e). In a second experiment the lung lobe was dissected and the small lobe section containing radioactivity was cut in 20 µm slices and their radioactivity monitored. All the radioactivity was found in a single slice with negligible radioactivity in the rest of the lung. Autoradiography of the radioactive slice and immediately adjacent sections showed a single radioactive spot in the ‘hot’ slice only, which establishes beyond doubt the presence of a single ^68^Ga-smSiP in the lung (Fig. 3f). The radioactive signal was quantified at different steps of the protocol, from the injection to the autoradiography step. The quantification of the injected dose and the activity in the lungs after the ex vivo biodistribution was carried out by gamma counting using a calibration curve with ^68^Ga standards (Supplementary Fig. 8). In the same manner, a calibration curve was performed with ^68^Ga standards for the quantification of the lung slice in autoradiography (Supplementary Fig. 9). The quantitative data, decay-corrected to the injection time, are consistent with the presence of a single hotspot containing all the radioactivity during the different steps of the in vivo protocol with an average deviation of 8.5 ± 2.2% between the gamma counting and autoradiography quantification (Fig. 3g).

### Real-time particle tracking (PEPT)

**Fig. 4:**
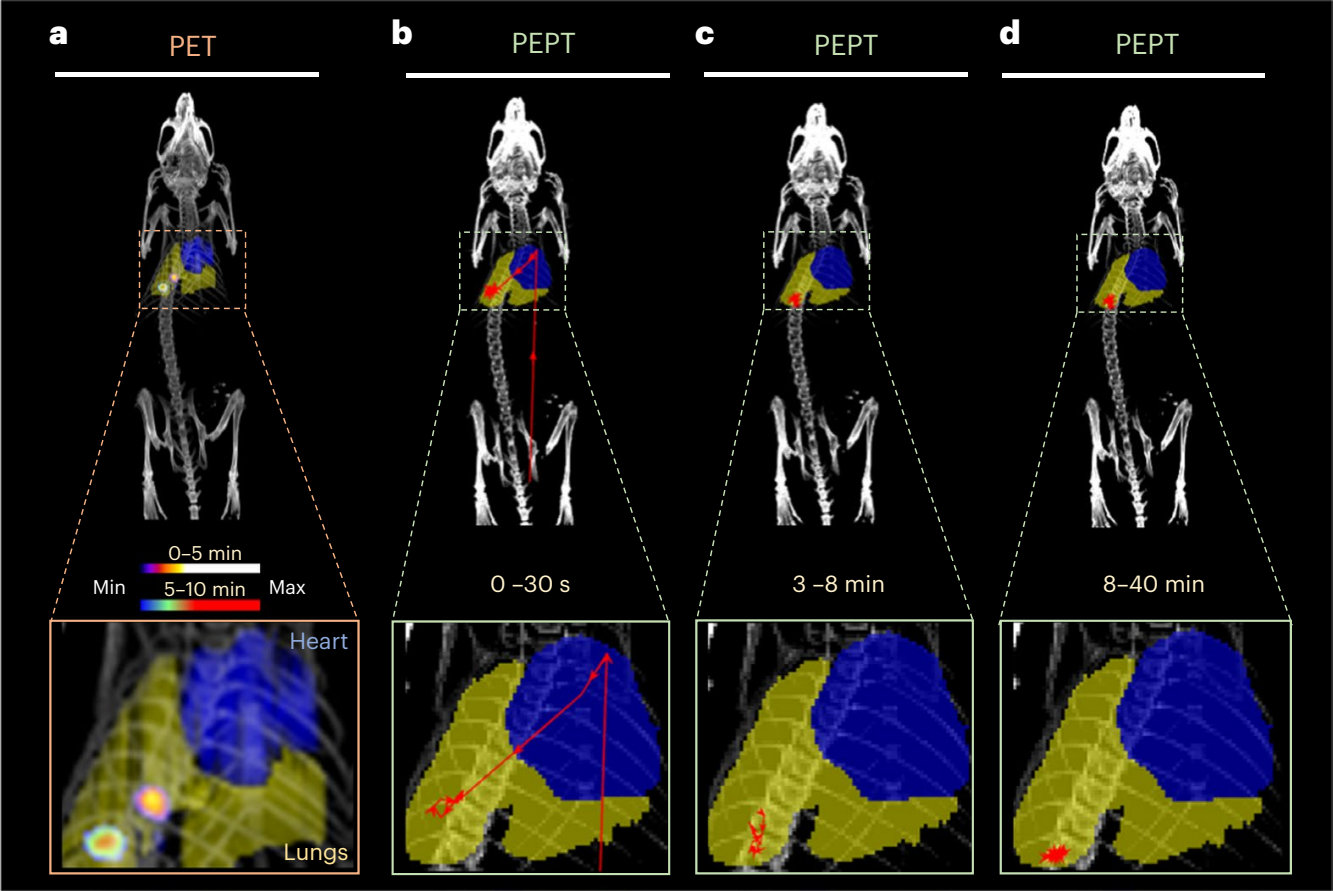
Comparison PET versus PEPT. **a**, Superimposed in vivo PET/CT images of a BALB/c mouse (same as in Fig. 3) at 0–5 min and 5–10 min after intravenous injection of a single 1.5 kBq ^68^Ga-smSiP with regions of interest (ROIs) drawn and represented to delimit the lungs (yellow) and the heart (blue). **b**–**d**, PEPT technique applied to a mouse using the listmode data generated from the PET acquisition. Lungs are delimited in yellow and the heart in blue with the red arrows depicting the real-time trajectory of the particle for whole-body and magnified image of coronal maximum intensity projections (MIP) at 0–30 s after particle injection (**b**), 3–8 min after particle injection (**c**) and 8–40 min after particle injection (**d**). The number of tracked points is reduced in the magnified coronal MIP to ease the interpretation of the particle movement.

Using the list-mode datasets originated through the PET acquisitions, the particle position was tracked using the Birmingham method for PEPT^16^. Figure 4a shows the PET images with the particle in the lungs in the first 5 min and moving position towards an inferior right part of the lung between 5–10 min post administration. Figure 4b–d shows the tracking of the particle at different timepoints, with the particle position registered to the CT (lungs are delimited in yellow and heart in blue for the sake of clarity; see supporting video^17^). Immediately following injection in the tail vein, the particle motion was rapid. Within the first 30 s of the scan, the first tracking point for the particle can be seen first in the lower abdomen; subsequently, the tracking reveals it travels through the heart and moves to the inferior region of the lungs (Fig. 4b). After the administration, the particle speed was estimated as approximately 48 mm s^−1^. Then, the particle moves between 3 min and 8 min after the administration to an inferior right part of the lung (Fig. 4c) remaining in this region for the rest of the scan (Fig. 4d). The anterior–posterior movement of the particle once within the lungs was confined to approximately ±2 mm, suggesting that the motion followed the breathing pattern of the mouse. In the rest of the mice, PEPT analysis shows a similar trajectory from the lower abdomen to the heart then to the lungs (Supplementary Fig. 10; see Supplementary Table 1 for links to videos).

### PEG_5k_-coated sub-micrometre-size silica particles

**Fig. 5:**
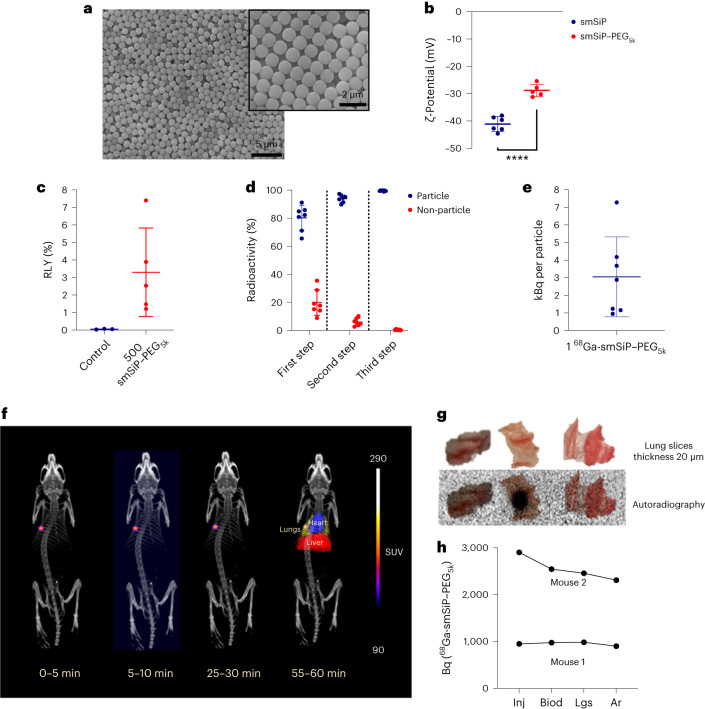
Physicochemical, radiochemical properties, and in vivo imaging of ^68^Ga-smSiP–PEG_5k_. **a**, Representative SEM micrographs of smSiP–PEG_5k_ at two different magnification scales (left image scale bar, 5 µm; right image scale bar, 2 µm). **b**, *ζ*-Potential measurements for smSiP (*n* = 6) and smSiP–PEG_5k_ (*n* = 5) resuspended at 1 mg ml^−1^ in distilled H_2_O, ****P < 0.0001, Student’s *t*-test. **c**, RLY for the control reaction (reaction without particles, *n* = 3) and 500 ^68^Ga-smSiP–PEG_5k_ (*n* = 5). **d**, Percentage of radioactivity in the ^68^Ga-smSiP–PEG_5k_ particle and non-particle fractions measured by gamma counter after the first (left), second (middle) and third (right) fractionation steps showing the difference in percentage of radioactivity between the single ^68^Ga-smSiP–PEG_5k_ fraction and the other three fractions (*n* = 7). **e**, Specific radioactivity (kBq) for the isolated (single) ^68^Ga-smSiP–PEG_5k_ (*n* = 7). **f**, In vivo PET/CT imaging of a BALB/c mouse at 0–5, 5–10, 25–30 and 55–60 min after intravenous injection of a single 2.9 kBq ^68^Ga-smSiP–PEG_5k_. Regions of interests (ROIs) were drawn and represented in 55–60 min to delimit the lungs (yellow), the heart (blue) and the liver (red) for better clarity. Scale bar represents standardised uptake value (SUV). **g**, Autoradiography of 20 µm slices of the lung. The radioactivity was monitored for each slice and the autoradiography carried out on the ‘hot’ slice and the two slices adjacent to it. Radioactive signal, represented in black within the tissue slices, above background was only found in the ‘hot’ slice. **h**, Quantification of the radioactivity in Bq for the different steps involved during the in vivo experiments for mouse 1 injected with 0.95 kBq of ^68^Ga-smSiP and mouse 2 injected with 2.9 kBq. All the measurements are decay-corrected to the time of injection for better comparison. Inj, activity measured in a gamma counter before the injection; Biod, activity in the lungs measured by gamma counter during the biodistribution studies; Lgs, activity in the 20 µm lung tissue slice measured by gamma counter; Ar, activity in the 20 µm lung tissue slice measured by autoradiography. Data are presented as mean ± s.d. Source data

**Fig. 6:**
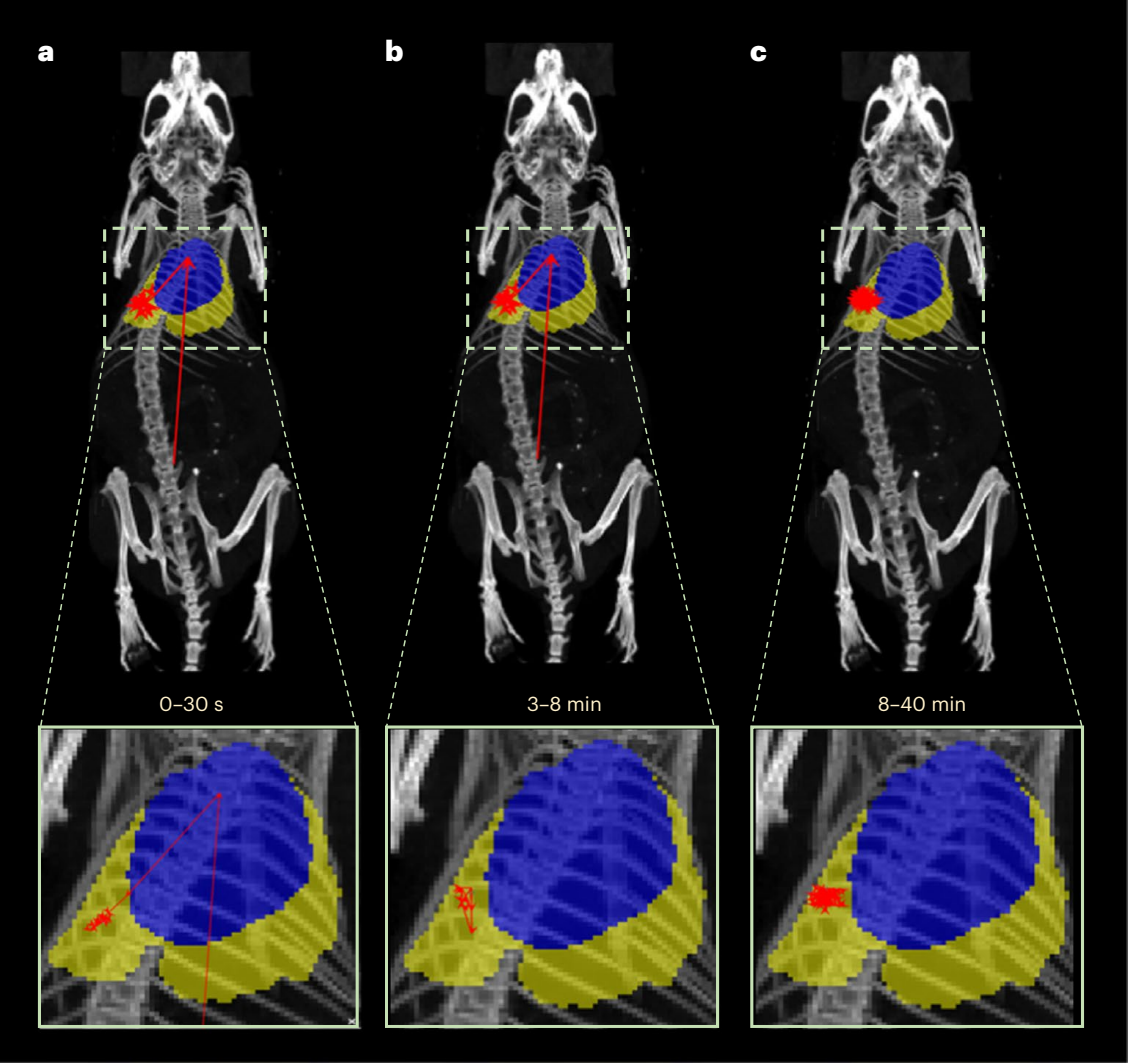
PEPT reconstructions for ^68^Ga-smSiP–PEG_5k_. **a**–**c**, PEPT technique applied to a mouse using the listmode data generated from the PET acquisition after intravenous injection of a single 2.9 kBq ^68^Ga-smSiP–PEG_5k._ Lungs are delimited in yellow and the heart in blue with the red arrows depicting the real-time trajectory of the particle for whole-body and magnified image of coronal maximum intensity projections (MIP) at 0–30 s after particle injection (**a**), 3–8 min after particle injection (**b**) and 8–40 min after particle injection (**c**). The number of tracked points is reduced in the magnified coronal MIP to ease the interpretation of the particle movement.

To investigate whether the lack of coating of the particle is responsible of the rapid uptake of the smSiP in the lung, further experiments with PEGylated particles were conducted. The uncoated particles were grafted with a mPEG_5k_–silane with a reaction yield of 43% providing 54 molecules of mPEG_5k_ per silica particle. SEM images of the PEG_5k_-coated sub-micrometre-size silica particles (smSiP–PEG_5k_) did not reveal any changes in shape and size compared with the uncoated particles (Fig. 5a). *ζ*-Potential measurements revealed an increase in the surface charge from −41.1 ± 3.3 mV for smSiP to −28.4 ± 3.1 mV for smSiP–PEG_5k_ (Fig. 5b). Radiolabelling reactions with ^68^Ga were conducted for 500 smSiP–PEG_5k_ following the same conditions as with smSiP, providing an RLY of 3.3 ± 3.1% (Fig. 5c). In this case, the isolation of a single ^68^Ga-smSiP–PEG_5k_ particle required three fractionation steps to obtain an RCP >99% (Fig. 5d) with a specific activity of 3.1 ± 3.2 kBq per particle (Fig. 5e). In vivo experiments in healthy Balb/c mice (*n* = 2) with 0.95–2.9 kBq of ^68^Ga-smSiP–PEG_5k_ showed a single hotspot in the lungs that remains static for the length of the scan (Fig. 5f and Supplementary Fig. 11). Quantification for each time frame of the reconstructed images revealed a mean value of 96.9 ± 2.8% of the injected activity (Supplementary Fig. 12) in the lungs and the ex vivo biodistribution studies showed the absence of radioactivity in other organs (Supplementary Fig. 13). As with smSiP, the single hotspot was identified in a 20 µm lung tissue slice with no radioactivity detected in the rest of the organ (Fig. 5g). Finally, the quantification of the radioactive signal during the different steps, from injection to the autoradiography corrected to the time of injection, evidenced the presence of a single source of radioactivity providing similar quantitative values from all the techniques with an average deviation of 14.8 ± 6.6% (Fig. 5h). As expected based on the PET/CT imaging results, further PEPT analysis confirmed that the coating did not allow the particle to circulate beyond the lung (Fig. 6 and Supplementary Fig. 14; see Supplementary Table 1 for links to the videos).

## Discussion

The development of suitable single-particle tracers is the main challenge that has restricted the exploration of PEPT for in vivo applications. To address this, we synthesized extremely homogeneous non-porous silica particles of 950 ± 50 nm diameter. The choice of the particle size and material is not trivial: with higher activities per particle expected for larger particles, a size between 900 and 1,000 nm was specifically targeted to obtain the largest radioactivity per particle while minimizing the risks associated with intravenous administration of particulate materials. The selected material, silica, presents high stability and biocompatibility in vivo and is also well known to provide excellent radiolabelling with a variety of oxophilic radiometals such as ^68^Ga while the integrity of the particle is not affected by the radiolabelling reaction^9,15^.

To isolate a single particle, a precise method to control the number of particles during the radiolabelling was lacking. The physicochemical properties of smSiP enable the use of flow cytometry. The small size of the particles, compared with cells, resulted in large deviations in the calculated number of particles after sorting^18^. However, it enabled precise and consistent particle quantification allowing the optimisation of the radiolabelling reaction for as few as 500 particles. For radiolabelling, a three-step purification protocol based on decreased concentrations of EDTA (a known trivalent metal chelator) was implemented for the removal of unreacted and colloidal ^68^Ga. The RLY of 1.9 ± 1.3% achieved can be considered very low in general terms of radiolabelling a substance, but it is remarkably high considering that the radiolabelling involved only 500 nanoparticles (radiolabelling reactions are usually conducted for 10^9^–10^12^ molecules and 10^6^–10^9^ particles). The high RLY facilitated isolation of a single particle using a simple fractionation protocol. With the fractionation protocol, the formation of colloidal aggregates during the radiolabelling became discernible since sonication between isolation steps leads to increased radioactivity levels in all fractions due to aggregate desorption. Thus, aggregates can be easily excluded, and the single particle isolated in the fraction displaying consistent levels of radioactivity distributed uniformly between fractionation steps following sonication. The method provided a specific activity of 2.1 ± 1.4 kBq per particle, which is ca. 100 times higher than the radioactivity reported for a single cell and much higher than any reported nanoparticulate radiotracer so far^7^.

PET/CT images show the single particle clearly after 5 min post administration confirming the high RCP and in vivo stability. The high image quality after only 5 min with kBq levels of radioactivity (instead of MBq levels for standard PET tracers; ca. three orders of magnitude difference) is most probably due to the high specific activity of the particle, its pinpoint nature and the static position in the lung. The rapid lung uptake could be attributed to the uncoated nature of the silica particle. This might promote the formation of a protein corona in blood, increasing the size and restricting the flow through the lung capillaries^19^. To test this hypothesis, the particles were grafted with a PEG_5k_–silane conjugate. The reactivity of silane coupling agents with silica materials is commonly employed to attach different functional groups or biomolecules onto silica particles^20^. The coated particles required an extra fractionation step to isolate a single particle which might be due to unspecific interactions between the PEG_5k_ and ^68^Ga that release weakly bound ^68^Ga from the particle during the fractionation steps. In vivo experiments revealed a similar behaviour with a rapid uptake in the lung. We suggest that this may be the result of two main factors: the size/rigidity of the particle or the fact that it is a single particle, which makes it highly susceptible for phagocytosis. Mouse lung capillaries have been reported to be as small as 2 µm in diameter, representing a size exclusion filter for blood-circulating components^21^. However, unlike silica, which is a rigid material, blood cells are deformable and sense the mechanical forces imposed by the lung capillaries to change their shape and continue flowing^22,23^. Besides, the unique nature of being a single particle increases the statistical probability of being engulfed by phagocytic cells within the lung. In particular, neutrophils are abundant in the lung, forming intravascular reservoirs known as lung-marginated neutrophil pools^24^. It is plausible that our single particles are promptly recognized and engulfed by these pools or other phagocytic cells, presenting another potential explanation for rapid accumulation in the lung. To address these issues in the future, our research will delve into the exploration of smaller and, most importantly, flexible particles and investigate strategies to temporarily inhibit the phagocytic response of lung neutrophils.

Real-time tracking PEPT results are in agreement with those observed in the PET images, providing confidence that its implementation on the pre-clinical scanner was successful. PEPT yields additional information not available from conventional PET. We successfully quantified particle velocity within the initial moments following particle administration. At the later timepoints, the motion quantification in the anterior–posterior direction allowed the measurement of the breathing motion in mice^25^. Overall, the trajectory was consistent, suggesting a high degree of repeatability in the experimental methods. It is important to note that the temporal resolution of PEPT is contingent upon the number of time-consecutive LoRs (often referred to as LoR sample size) used for calculating each tracking point. In this work, the LoR sample size was varied between 1 s and 5 s to capture rapid particle motion at earlier tracking points, while later points were sampled at approximately 30–60-s intervals to improve positioning error. Consequently, the positioning error for the earlier points exhibited an increased deviation of ±1.9 mm, whereas the later points displayed a smaller deviation of 0.9 mm.

In conventional PET, the high positron range of ^68^Ga leads to worsening spatial resolution when compared with ^18^F-based radiotracers. Using the Birmingham method, the position of a particle at a given time is calculated using the minimum distance point (MDP) between the LoRs. Since positrons are emitted isotropically, the MDP (hence the position of the particle) should not be affected by positron range in a homogeneous medium. However, there are contrasting tissue densities in a mouse notably near lung–tissue interfaces, and this may introduce a bias in the calculated position of the particle. Although previous PET imaging studies have shown that for ^18^F the positron range profiles in the lung are very similar to soft tissue, higher energy positron emitters exhibit greater difference. Indeed, the bias will increase with increasing positron energy but will depend among other factors on source size and local tissue density. Future evaluations are required to assess the magnitude of the bias with different positron emitters and PEPT algorithms. Another possible limitation includes the well-known intrinsic background count rate that occurs in PET scanners using cerium doped lutetium yttrium orthosilicate scintillation crystals (LYSO:Ce). Intrinsic coincidences produced by LYSO can be misinterpreted as true coincidences and because these are distributed uniformly in the field of view of the scanner could increase the error in positioning. To minimize the influence of the intrinsic LYSO coincidences, we set the lower level discriminator of the energy acquisition window to 400 keV; previous work has shown that at this level the intrinsic count rate is negligible^26^. In addition, any random coincidences due to the intrinsic background are rejected before applying the PEPT tracking algorithm. Furthermore, it is not clear if other available algorithms will produce improved results since the experiments presented in this work operate in a different parameter envelope (for example, activity used, particle speeds and so on) to industrial applications. For instance, one would expect a curved particle trajectory through the inferior vena cava to the heart; however, only two positions between could be calculated, given the activity and speed of the particle, with reasonable error yielding an apparent straight-line trajectory. Although this has not impacted the overall feasibility of the method, we are currently performing further evaluation to assess the best tracking technique for small animal experiments.

## Conclusions

We have developed a single radiolabelled particle for in vivo nuclear imaging and real-time particle tracking. From the synthesis of highly homogeneous sub-micrometre size particles to the radiolabelling method optimized for a minimal number of particles, and the single particle isolation, these notable challenges have been successfully addressed. Importantly, the reported protocols provide a single, highly pure radiolabelled particle with outstanding specific activity and quantitative RCS. PET imaging studies showed high-quality images after the administration of the single particle even with low radioactive doses (0.4–2.9 kBq), which are approximately three orders of magnitude lower than those required for conventional PET tracers, and in a short time window (0–5 min). Furthermore, the implementation of a reconstruction protocol as well as the optimization of the Birmingham PEPT algorithm to our pre-clinical data have made it possible to track the single particle as it moves within mice. Notably, the tracking of the particle provides important valuable information such as the particle velocity, directly related with the blood flow and pressure differentials, or the breathing motion. As a proof of concept of the implementation of real-time tracking in pre-clinical imaging, these initial results are certainly promising for the evaluation of the blood flow in restricted regions such as the lung capillaries, in diseases derived from cardiovascular conditions, and in new vessels and shunts triggered by a tumoral growth, all of which are difficult to study through conventional methods in a quantitative manner. Diagnostically, PEPT has the potential to provide new insights on different conditions related to blood flow such as stenosis, thrombosis, atherosclerosis and angiogenesis, among others, by tracking the trajectory and velocity of particles in blood vessels. Furthermore, PEPT holds a substantial potential in assessing organ motion, particularly within tumours, as well as enabling tumour tracking alongside complementary methodologies such as PET-guided radiotherapy or radioguided surgery. Importantly, as it requires a single radiolabelled nanometric particle, the amount of material and radioactivity to administer is minimal with negligible toxicity issues that anticipate a high potential for translation of single particle tracers to clinical evaluation. In vivo PEPT is particularly timely considering the advent of total-body clinical PET scanners. With increased sensitivities and the possibility of tracking the particle in a whole-body field of view within the patient, the combination of PEPT and total-body PET is certainly an exciting opportunity to quantitatively study the complexity of blood flow and cell margination in real time and at the whole-body level.

## Methods

All reagents were used as received unless stated otherwise. All chemicals were purchased from Sigma Aldrich except for the counting beads (CountBright Absolute Counting Beads, Invitrogen). *ζ*-Potential was measured using a Zetasizer NanoZS90 (Malvern Instruments). Size and morphology of the particles was studied by SEM in a JEOL JSM 7800F Prime microscope with integrated EDS to provide the elemental analysis. Particle size was determined by measuring 50 independent particles. Radio instant thin-layer chromatography (ITLC) was developed on Agilent Technologies glass microfibre chromatography paper impregnated with silicic acid and analysed using a Lablogic Flow-count TLC scanner and a BioScan B-FC-3200 photomultiplier tube (PMT) detector using Laura software. The ITLC mobile phase was composed of 0.175 M citric acid and 0.325 M trisodium citrate in water unless stated otherwise. Radioactive samples were measured using a Capintec CRC-25R (Capintec) or an LKB Wallac 1282 Compugamma CS (PerkinElmer) for which data were collected using EdenTerm software. Flow cytometry experiments were carried out in a BD FACSMelody cell sorter using BD FACSChorus Software. PET/CT images were acquired using a NanoPET/CT scanner (Mediso), reconstructed using Nucline v.0.21 software, and images were analysed using VivoQuant software (version 3.5, InviCRO). Listmode data were obtained by a specific MATLAB software tool developed by Mediso. Autoradiography was performed in a GE Amersham Typhoon instrument.

### Synthesis of sub-micrometre-size silica particles

The particles were synthesized using the Stöber method. This method is based on the hydrolysis and consecutive condensation of silicon alkoxides to produce monodisperse, spherical silica particles^27^. Tetraethyl orthosilicate (TEOS) was used as silicon source, ammonia as base catalyst and potassium chloride as electrolyte. A solution of TEOS in ethanol was continuously added to a solution containing the catalyst and the electrolyte. Modification of the reagent starting quantity or addition rate provides differences in the particle size as previously reported^28^. Here, two solutions were prepared before the synthesis of the particles: Solution 1 containing 19.0 mmol of TEOS in 33.3 ml of EtOH and Solution 2 containing 0.23 mmol of KCl in 9 ml of ammonia, 65 ml of EtOH and 6.75 ml of H_2_O. For the synthesis, Solution 2 was placed in a 250 ml round-bottom flask heated at 50 °C under stirring at 300 rpm for 15 min. Then, Solution 1 was added dropwise to Solution 2 (supply rate 0.2 ml min^−1^). After addition of Solution 1, the obtained particles were purified by centrifugation at 18,300*g* for 3 min and washed with EtOH five times. Finally, the SiO_2_ microparticles were dried under vacuum.

### Grafting of the sub-micrometre-size particles with silane–PEG_5k_

A 20 mg ml^−1^ solution of silane–PEG_5k_ (Sigma Aldrich) in EtOH 98% was added over a solution of smSiP at 5 mg ml^−1^ in EtOH 98% and 2.8% of ammonia. The mixture was stirred overnight at room temperature, and the particles were recovered by centrifugation at 18,300*g* for 3 min. Finally, the particles were washed three times with distilled water and dried under vacuum overnight. The washing solutions were freeze-dried overnight and the amount of unattached silane–PEG_5k_ weighted for the reaction yield calculation. A 0.05 mg ml^−1^ solution of smSiP–PEG_5k_ in distilled water was employed for further radiolabelling reactions.

### [^68^Ga]GaCl_3_

Gallium-68 was eluted as [^68^Ga]GaCl_3_ from an Eckert and Ziegler ^68^Ge/^68^Ga generator in ultrapure HCl (4 ml, 0.1 M) manufactured to good manufacturing practice requirements (ABX).

### Concentration of the [^68^Ga]GaCl_3_ elution by cation exchange

The concentration of the elution was carried out using the setup described in Supplementary Fig. 1. First, the 4 ml of the [^68^Ga]GaCl_3_ elution were loaded onto a Strata-X-C 33u cartridge (Phenomenex) and the eluate was discarded. Then, the cartridge was washed with 5 ml of an acetone/0.1 M HCl (80:20) solution and the eluate was discarded. Finally, the concentrated [^68^Ga]GaCl_3_ was collected by adding 700 µl of an acetone/0.05 M HCl (98:2) solution, dried under a N_2_ stream and resuspended in 50 µl of 0.5 M HEPES buffer, (pH 4.9). Radio-TLC was performed at the different stages for quality control. The protocol takes approximately 20 min providing a recovery yield of 86.2 ± 8.5%.

### Radiolabelling of silica particles at different concentrations with ^68^Ga

Silica particles were resuspended at different concentrations (from 1 to 0.002 mg ml^−1^) in 0.5 M HEPES buffer (pH 4.9). Then, 50 µl of the solution was added into a reaction tube before the addition of the concentrated [^68^Ga]GaCl_3_ elution in 50 µl of 0.5 M HEPES buffer (pH 4.9). Reactions were conducted at 90 °C for 30 min, and radio-TLC was carried out to calculate the radiochemical yield.

### Measurement of particle concentration by flow cytometry

Particle concentrations were calculated by flow cytometry using counting beads (CountBright Absolute Counting Beads, Invitrogen) following the manufacturer’s instructions. Silica particles were resuspended at 0.05 mg ml^−1^, sonicated for 10 min and passed through a 10 µm cut-off size filter (KX Syringe Filter, Nylon, 25 mm, 10 µm). The CountBright Absolute Counting Beads were warmed to room temperature and vortexed for 30 seconds. Then, 50 µl of beads were added to 300 µl of silica particles and the mixture was vortexed for 30 min to obtain a homogeneous solution. The sample was run on the flow cytometer and the forward scatter (FSC) threshold set to include the beads and the particles on the linear-FSC versus linear-side scatter plot. Afterwards, the fluorescence detector voltage was adjusted for the counting beads and a gating strategy performed to isolate the silica particles and the counting beads populations. Finally, gates on the particles and the absolute counting beads were drawn and 1,000 bead events were recorded for each sample. Using this strategy, the number of particles in solution was calculated using the following equation:

$$\begin{array}{l}\displaystyle{\mathrm{Absolute}}\,{\mathrm{count}}\,\left(\frac{\mathrm{Particles}}{{{\upmu l}}}\right)=\displaystyle\frac{({\mathrm{Particles}}\,{\mathrm{count}}\,\times\,{\mathrm{Counting}}\,{\mathrm{beads}}\,{\mathrm{volume}})}{({\mathrm{Counting}}\,{\mathrm{beads}}\,{\mathrm{count}}\,\times\,{\mathrm{Particles}}\,{\mathrm{volume}})} \times\,{\mathrm{Counting}}\,{\mathrm{beads}}\,{\mathrm{concentration}}\left(\frac{{\mathrm{Beads}}}{{{\upmu l}}}\right)\end{array}$$

### Radiolabelling of 500 smSiP

Five-hundred smSiP were added to 50 µl of the concentrated [^68^Ga]GaCl_3_ elution in 0.5 M HEPES buffer pH 4.9. Then, 5.6 µl of polysorbate 80 was added and the mixture was heated at 90 °C for 30 min at 900 rpm in a thermal mixer. Afterwards, a final multi-step purification protocol was designed to remove unreacted/colloidal ^68^Ga. Fifty microlitres of 10 mM EDTA were added, and the mixture was incubated 5 min at room temperature. Then, the particles were centrifuged for 3 min at 18,300*g*, resuspended in 500 µl of PBS containing 1 mM EDTA + 0.1% polysorbate 80 and gently vortexed for 10 s. The particles were centrifuged again, washed with a solution of 0.1 mM EDTA + 0.1% polysorbate 80 in PBS and gently vortexed for 10 s. Finally, the particles were centrifuged and washed five more times with PBS + 0.1% polysorbate 80 and resuspended in 500 µl PBS. The radiolabelling reaction was monitored by radio-TLC during the successive reaction steps to evaluate the presence of colloids (that can be confused with particles if not removed properly), the radiolabelling of the particles and the purity of the final product. RLY was calculated by comparison between the amount of radioactivity in the particles and the supernatants after the washing steps.

### Fractionation

For the fractionation strategy, volumes from 0.5 µl to 20 µl of the ^68^Ga-smSiP at a theoretical concentration of 1 particle µl^−1^ were added into different sample tubes in 1 µl steps (0.5, 1, 2, 3…) and PBS was added to bring the final volume to 50 µl. Then, 37.5 µl from the first tube were pipetted into a second sample tube, 25 µl of the second tube into a third tube and finally 12.5 µl of the third tube to a fourth tube. This strategy provides four tubes per sample with a final volume of 12.5 µl per tube. The radioactivity in each tube was measured in a gamma counter and the values were calculated in kBq using a calibration curve, for further comparison and analysis. The samples containing most of the radioactivity in only one tube were sonicated for 30 s at room temperature and subjected to a second fractionation step. Then, the samples in which all the radioactivity was found in a single tube (with negligible activity in the other three tubes) were used for further in vivo/ex vivo experiments.

### PET/CT phantom imaging

A phantom imaging experiment was carried out with one ^68^Ga-smSiP. A cannula was used to deliver the particle into a sample tube to evaluate whether a single particle could remain trapped in the cannula tubing during administration. Briefly, the phantom tube was placed in the nanoPET/CT scanner with the end of the cannula tip attached to the tube. After starting the PET acquisition, the particle resuspended in 100 µl of PBS was delivered with an insulin syringe attached to the beginning of the cannula. Then, the cannula was washed with 50 µl of PBS to ensure the delivery of the particle into the phantom tube. The PET acquisition was carried out for 2 h followed by a standard CT scan.

### In vivo PET/CT imaging

Animal imaging studies were ethically reviewed and carried out in accordance with the Animals (Scientific Procedures) Act 1986 (ASPA) UK Home Office regulations governing animal experimentation. In vivo imaging was conducted in healthy 8-week-old BALB/c mice. Animals were anaesthetized with isoflurane (2–3% in oxygen), cannulated and placed on the scanner bed under anaesthesia. The bed was heated to 37 °C by internal air flow to keep the animal at normal body temperature, and the respiration rate was monitored and maintained at 60–80 breaths min^−1^ throughout the scan. Maintaining control over the animal temperature is important, as an unexpected drop in temperature could lead to a reduction in the velocity of the particle in blood. One ^68^Ga-smSiP (*n* = 4) or ^68^Ga-smSiP–PEG_5k_ particle (*n* = 2) was administered through the cannula in 100 µl of PBS, followed by wash with 50 µl PBS after starting the PET acquisition (1:5 coincidence mode; 5 ns coincidence time window). PET was recorded for 2 h, and then a semicircular CT scan was performed. Animal body temperature and respiratory rate were monitored during the whole process. Dynamic PET/CT images were reconstructed using Tera-Tomo 3D reconstruction (400–600 keV energy window, 1:5 coincidence mode, 20 iterations and 1 subset) at a voxel size of 0.4 × 0.4 × 0.4 mm^3^ and corrected for attenuation, scatter and decay. List-mode data for all PET/PEPT acquisitions can be found for ^68^Ga-smSiP at ref. ^29^ and for ^68^Ga-smSiP–PEG_5k_ at ref. ^30^.

### Real-time tracking

First, data were exported from the scanner in listmode format (that is, a format with a timestamp and crystal index for detected coincidence photons). A geometric transformation was applied to convert from crystal indices to position in mm units. The Birmingham method iteratively calculates the MDP from a subset of all the LoRs. It does this by discarding LoRs that are further than a set distance from the MDP as these are likely to arise from false LoRs, for example, LoRs that may originate from scatter. The MDP is refined with each iteration; the number of iterations is effectively set by the *f*-factor and relates to the total number of LoRs that are used to estimate the final particle position within that subset (for example, an *f*-factor of 0.5 means that the iteration loop will terminate when 50% of the LoRs in the subset remain). The number of LoRs used in a subset can be reduced to improve the temporal sampling (the subsets are time consecutive with no overlap) at the cost of increasing the uncertainty in position (further details of the algorithm can be found in Parker et al.^5^) The Birmingham method was used to analyse list-mode data from the PET scanner. An adaptive sample size was used to track the particle in the mice. The sample size was set to achieve a balance of sufficient temporal sampling while minimizing positioning errors. A sample size between 100 and 200 LoRs was used in the early stages of the scans (<60 s from scan start), with *f* = 0.1, yielding approximately 1–5 s intervals. At scanning times >60 s, sample sizes were varied between 1,000 and 2,000, which yielded time intervals of between 30 s and 60 s depending on the in vivo experiment. The number of counts used to calculate the MDP (in the final iteration) can be found by multiplying the sample size by the *f*-factor value. These parameters were based on prior experience and informed by previous publications^1^.

Speed was obtained as $\sqrt{{v}_{x}^{2}+{v}_{y}^{2}+{v}_{z}^{2}}$ where ${v}_{m}^{2}$ is the velocity in the *x*, *y* and *z* directions.

### Ex vivo organ uptake

Uptake in different organs was evaluated by gamma counting. After the in vivo PET/CT imaging, animals were killed by cervical dislocation and organs excised and weighed for radioactivity counting in a gamma counter (LKB Wallac 1282 Compugamma CS). Data were expressed as percentage injected dose (dose in the organ/total dose injected) per gram of tissue (%ID g^−1^).

### Autoradiography

The radioactivity in the lungs was traced with a radiation detector (EP15 probe, Morgan), and the lungs were cut into small sections with a scalpel until a small portion of tissue with the radioactive signal was obtained. The tissue was snap frozen in −80 °C isopropanol. Immediately after freezing, the tissue was embedded in optimal cutting temperature medium and cut in a cryostat in 20 µm slices. Each slice was surveyed with the detector until the radioactive slice was found. The previous (below background), radioactive and next (below background) slice were placed on a Superfrost microscope slide (Epredia). The rest of the remaining tissue was also below background. The microscope slide with the three sections was covered with cling film and opposed to a GE autoradiography plate overnight. The plate was analysed using GE Amersham Typhoon with 25 µm resolution and PMT setting of 4,000. The autoradiography image was superimposed on the picture of the tissue, showing one spot of radioactivity in the radioactive slice. For the quantification, standards were prepared in different known activities, and each was spotted as 1 µl quintet in paper. The spots were incubated in the same storage phosphor screen, BAS-IP MS (Multipurpose Standard) from GE as the single particles quantified. The image was acquired with the Amersham Typhoon 5 with the Control Software version 2.0 in the phosphor mode with a pixel size of 100 µm and a sensitivity of 4,000. The images were quantified with the software ImageQantTL v10.0-261 using the gel quantification toolbox. The spots were corrected by choosing a region immediately before or after the spot as a constant background. The resulting volume of the spot was used to calculate the Bq in the particle on the basis of the calibration curve.

### Statistics and reproducibility

For quantitative analysis, a minimum of three biological replicates were analysed excluding the in vivo data of ^68^Ga-smSiP–PEG_5k_ (*n* = 2). Data were analysed by ordinary one-way analysis of variance (ANOVA) with Dunnett’s multiple comparisons test and Student’s *t*-test. A *P* value <0.05 was considered statistically significant.

## Online content

Any methods, additional references, Nature Portfolio reporting summaries, source data, extended data, supplementary information, acknowledgements, peer review information; details of author contributions and competing interests; and statements of data and code availability are available at 10.1038/s41565-023-01589-8.

### Supplementary information

Supplementary Information Supplementary Figs. 1–14, Scheme 1 and Table 1.

Supplementary Data Raw data Supplementary Figs. 1–13.

### Source data

Source Data Fig. 1 Raw data radiolabelling reactions.

Source Data Fig. 2 Raw data flow cytometry and radiochemical properties.

Source Data Fig. 3 In vitro and in vivo raw data.

Source Data Fig. 5 Raw data characterization and radiolabelling smSiP–PEG_5k_.

## Data Availability

The data supporting the findings of this study are available within this article and its Supplementary Information. The videos generated from PEPT data and the list-mode data can be found in figshare.com. Links to the videos are provided in Supplementary Table 1 and links to the list-mode data can be found in Methods. Source data are provided with this paper.

## Code availability

The Birmingham Algorithm used for the PEPT reconstructions is publicly available in Github at https://github.com/uob-positron-imaging-centre/pept.
