# Supplementary material for: In vivo real-time positron emission particle tracking (PEPT) and single particle PET
Source: Nat Nanotechnol. 2024 Jan 19;19(5):668–76. doi: 10.1038/s41565-023-01589-8 (PMC11106003; doi:10.1038/s41565-023-01589-8)
Supplement: Supplementary file 1 — Supplementary Figs. 1–14, Scheme 1 and Table 1. [file 41565_2023_1589_MOESM1_ESM.pdf]

nature nanotechnology

Article

https://doi.org/10.1038/s41565-023-01589-8

In vivo real-time positron emission particle tracking (PEPT) and single particle PET

In the format provided by the
authors and unedited

Table of Contents

Supplementary Fig. 1│ζ-Potential measurements. 2

Supplementary Fig. 2│Concentration of the [^{68}Ga]$GaCl_3$ elution...2

Supplementary Fig. 3│Radio - Thin Layer Chromatography evaluation. 3

Supplementary Fig. 4│Radiochemical purity (RCP) of ^{68}Ga-smSiP. 3

Supplementary Scheme 1│Theoretical calculation number of particles. 4

Supplementary Fig. 5│ Radio-TLC evaluation for the radiolabelling of 500 smSiP. 4

Supplementary Fig. 6│In vivo PET/CT imaging of a single ^{68}Ga-smSiP. 5

Supplementary Fig. 7│Quantification of PET signal for a single ^{68}Ga-smSiP. 6

Supplementary Fig. 8│Calibration curve [^{68}Ga]$GaCl_3$. 6

Supplementary Fig. 9│Autoradiography quantification. 7

Supplementary Fig. 10│PEPT images for ^{68}Ga-smSiP. 7

Supplementary Fig. 11│In vivo PET/CT imaging of a single ^{68}Ga-smSiP-PEG_{5k}. 8

Supplementary Fig. 12│Quantification of PET signal for a single 68Ga-smSiP-PEG5k. 8

Supplementary Fig. 13│Biodistribution of ^{68}Ga-smSiP-PEG_{5k}. 9

Supplementary Fig. 14│PEPT images for ^{68}Ga-smSiP-PEG_{5k}. 9

Supplementary Table 1│Links to PEPT videos. 10

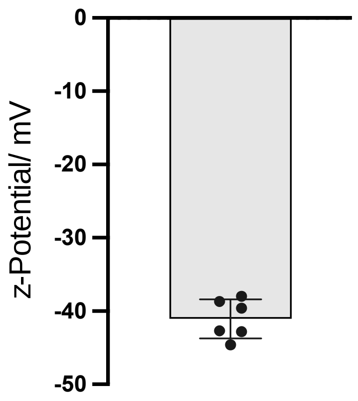

Supplementary Fig. 1 | ζ-Potential measurements. ζ-Potential represented as mean ± SD (n = 6) for smSiP resuspended at 1 mg/mL in distilled H_2O.

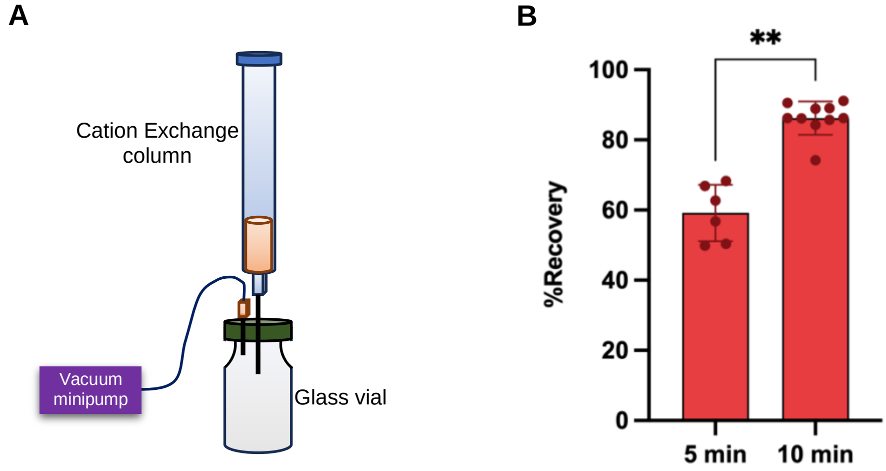

Supplementary Fig. 2 | Concentration of the [^{68}Ga]$GaCl_3$ elution. **A** Illustration of set-up used for the ^{68}Ga concentration by cation exchange, **B** %Recovery after concentration of the ^{68}Ga elution by cation exchange in 5 min (n = 6) and 10 min (n = 10, **p = 0.0015, two-tailed Student's *t*-test). Data are presented as mean ± SD

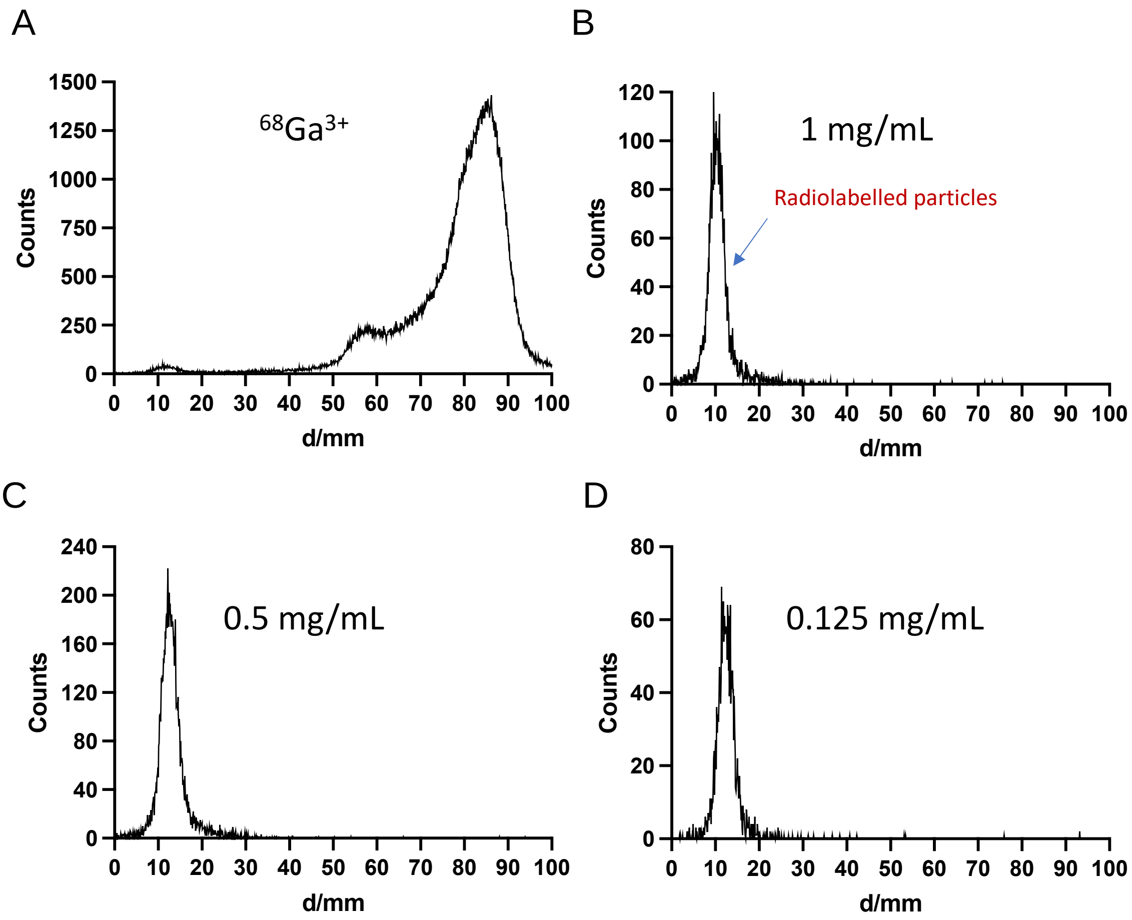

Supplementary Fig. 3│Radio - Thin Layer Chromatography evaluation. Radio-TLC chromatograms for **A**. "free $^{68}Ga^{3+}$", and after 30 min incubation at 90°C of ^{68}Ga with smSiP at **B.** 1 mg/mL, **C.** 0.5 mg/mL and **D.** 0.125 mg/mL.

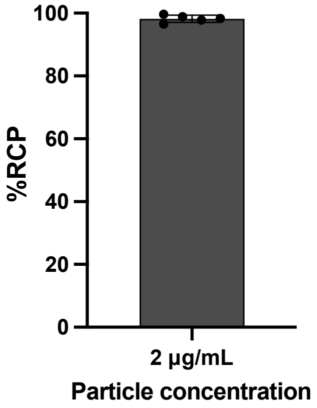

Supplementary Fig. 4│Radiochemical purity (RCP) of ^{68}Ga-smSiP. RCP for ^{68}Ga-smSiP at 2 µg/mL measured by radio-TLC after purification (mean ± SD, n = 5).

Theoretical calculation of number of particles:

Particle diameter = 950 nm

Particle radius (r) = 475 nm

Particle volume (V) = $\frac{4}{3}\pi r^3$ = 4.49 × 10^8 nm^3 = 4.49 × 10^{-13} cm^3

Density of silica (ρ_{SiO2} = 2.2 g/cm^3) = $\frac{m_{SiO2}}{V}$; m_{SiO2} = 9.88 x 10^{-13} g/SiO_2 particle

In 1 g of particles: $\frac{1\ \text{g}}{9.88 \times 10^{-13}\ \text{g/particle}}$ = 1.01 × 10^{12} SiO_2 particles

Supplementary Scheme 1│Theoretical calculation number of particles. Theoretical calculation of the number of SiO_2 particles with a density of 2.2 g/cm^3 assuming spherical shape of the particles.

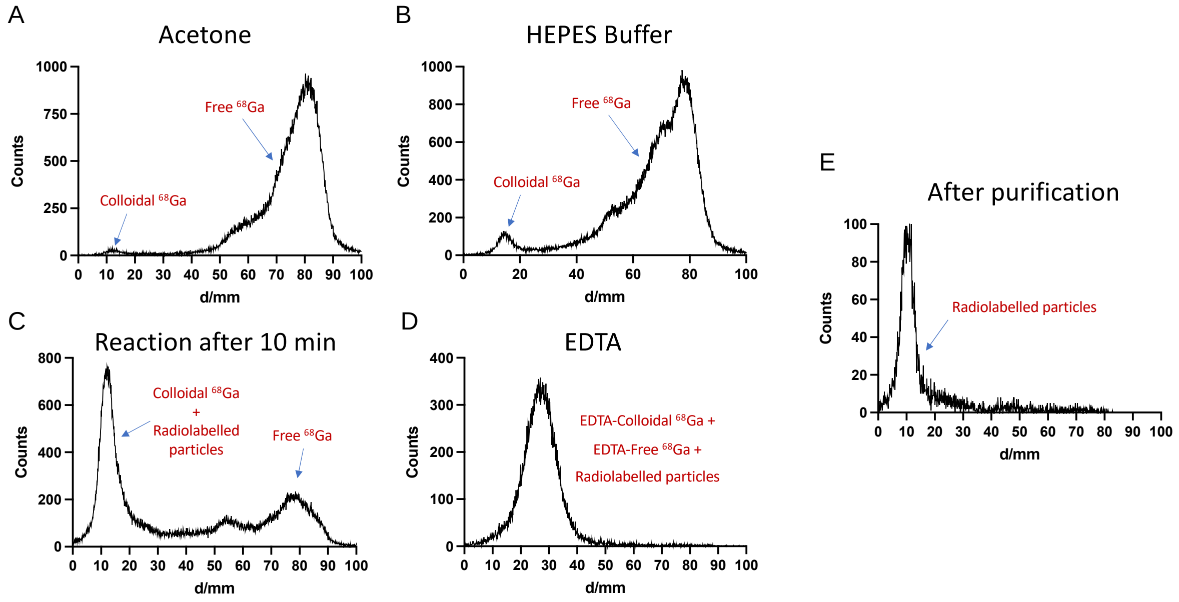

Supplementary Fig. 5 │Radio-TLC evaluation for the radiolabelling of 500 smSiP. A. After the elution of $^{68}GaCl_3$ in acetone from the cation exchange column. **B.** Resuspended $^{68}GaCl_3$ in 50 µL of HEPES 0.5 M pH = 4.7 previous to the reaction with the smSiP. **C.** 10 minutes after the reaction between $^{68}GaCl_3$ and smSiP, **D.** After the addition of 50 µL of 10 mM EDTA, **E.** After the purification protocol.

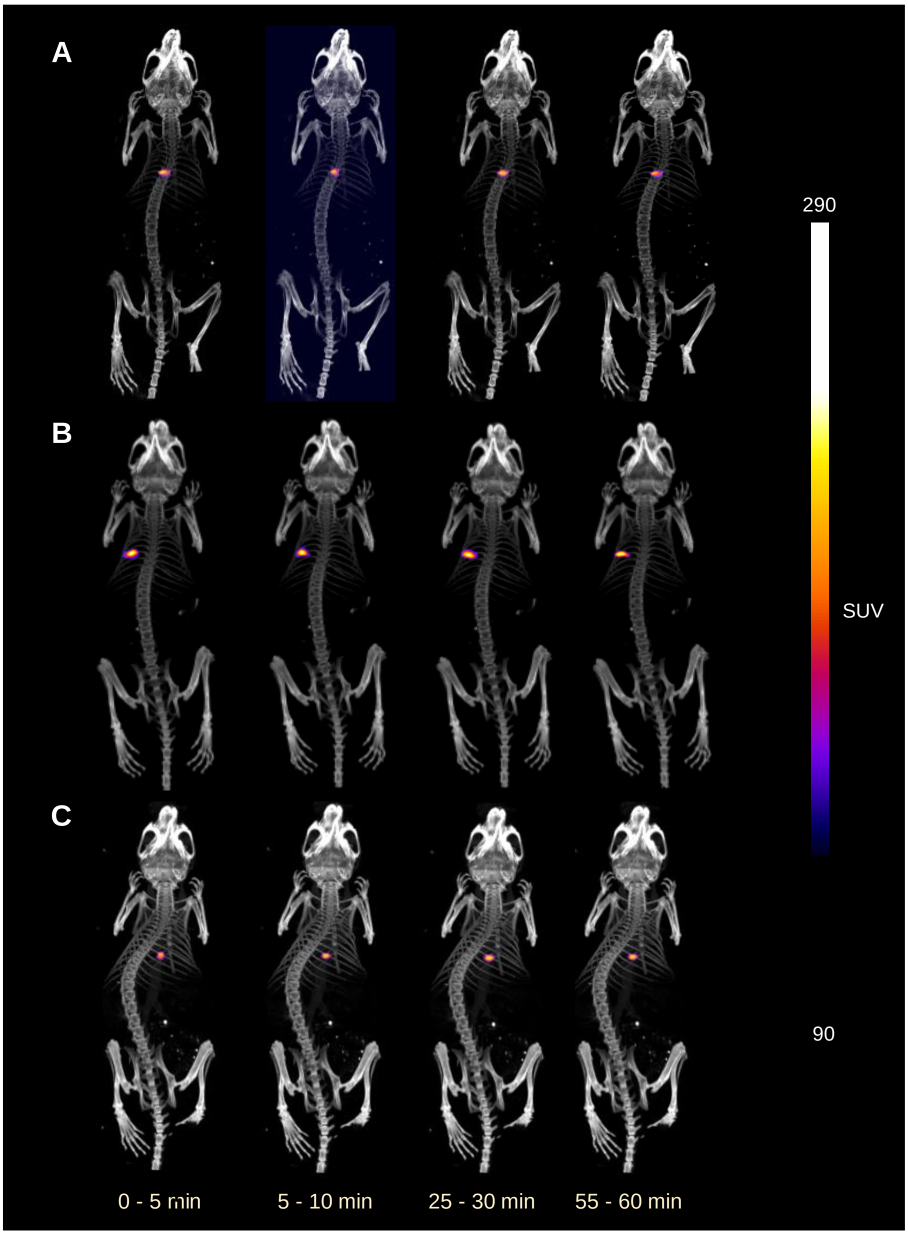

Supplementary Fig. 6│*In vivo* PET/CT imaging of a single ^{68}Ga-smSiP. *In vivo* PET/CT imaging of BALB/c mice (n = 3) at 0 - 5, 5 - 10 and 25 - 30 min after the *i.v.* injection of **A.** 1.1 kBq of ^{68}Ga-smSiP, **B.** 0.42 kBq of ^{68}Ga-smSiP and **C.** 1.9 kBq of ^{68}Ga-smSiP.

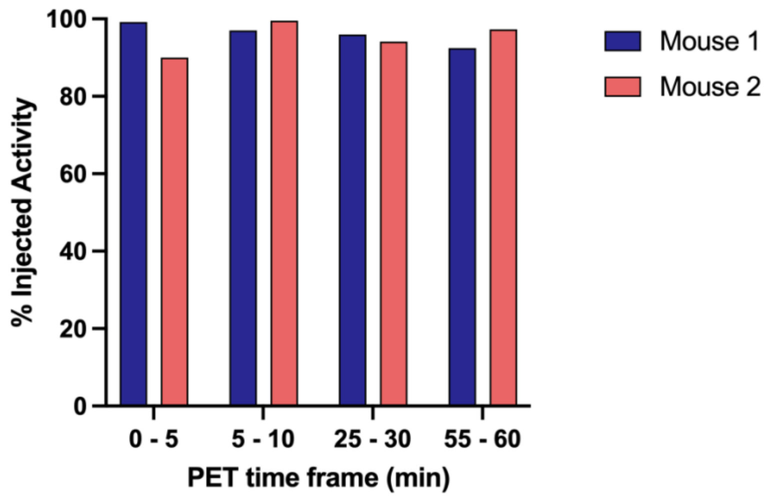

Supplementary Fig. 7 │ Quantification of PET signal for a single ^{68}Ga-smSiP. Quantification of the PET signal represented as % of injected activity for each time frame of the PET reconstruction for mouse 1 injected with 1.9 kBq of ^{68}Ga-smSiP and mouse 2 injected with 0.42 kBq of ^{68}Ga-smSiP.

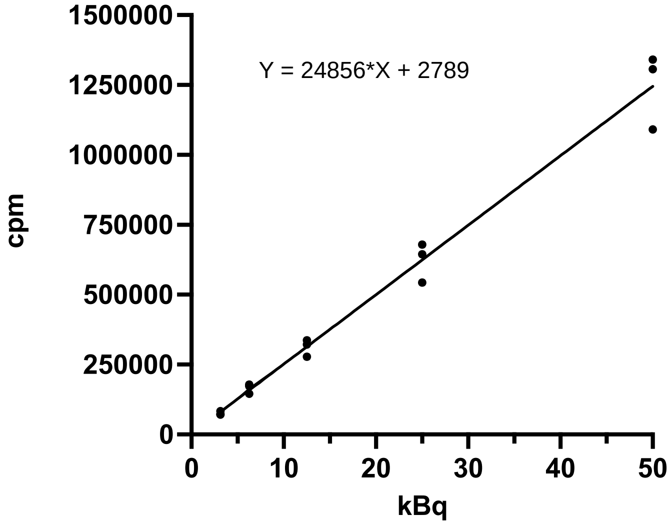

Supplementary Fig. 8│Calibration curve [^{68}Ga]GaCl$_3$. Calibration curve obtained after the representation of the quantified radioactive signal in the gammacounter (cpm) vs the amount of radioactivity measured in the activemeter for different ^{68}Ga standards (n = 3). Data are presented as individual values. Line represents the linear regression.

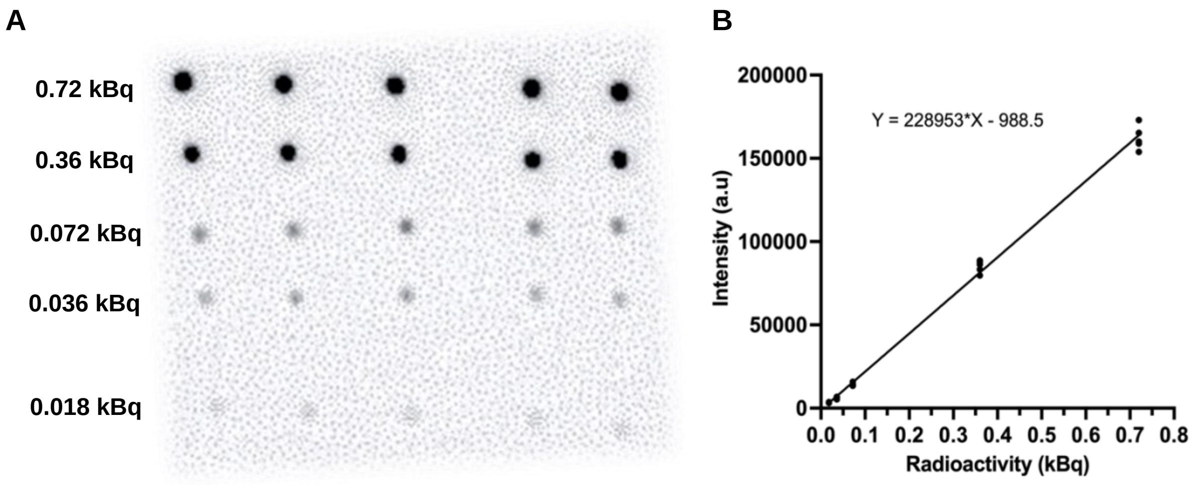

Supplementary Fig. 9 │ Autoradiography quantification. Calibration curve for autoradiography quantification, **A.** Autoradiography image obtain after the incubation for 16 h of 0.72, 0.36, 0.072, 0.036 and 0.018 kBq of $^{68}GaCl_3$ spotted in 1 µL (n = 5), **B.** Calibration curve obtained after the representation of the quantified autoradiography signal intensity vs the initial amount of radioactivity (n = 5), data are presented as individual values. Line represents the linear regression.

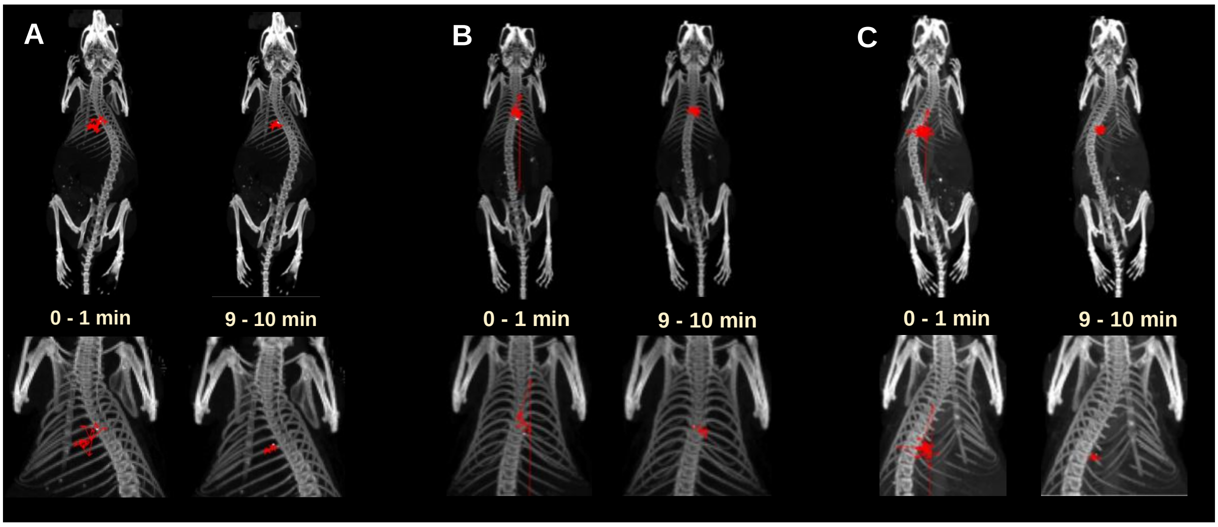

Supplementary Fig. 10│PEPT images for ^{68}Ga-smSiP. PEPT technique applied to Balb/c mice using the listmode data generated from the PET acquisition. Red arrows depict the real-time trajectory of the ^{68}Ga-smSiP particle for whole-body and magnified image of coronal MIP at: 0 – 1 min and 9 – 10 min after the injection of: **A.** 1.1 kBq of ^{68}Ga-smSiP, **B.** 0.4 kBq of ^{68}Ga-smSiP and **C.** 1.9 kBq of ^{68}Ga-smSiP.

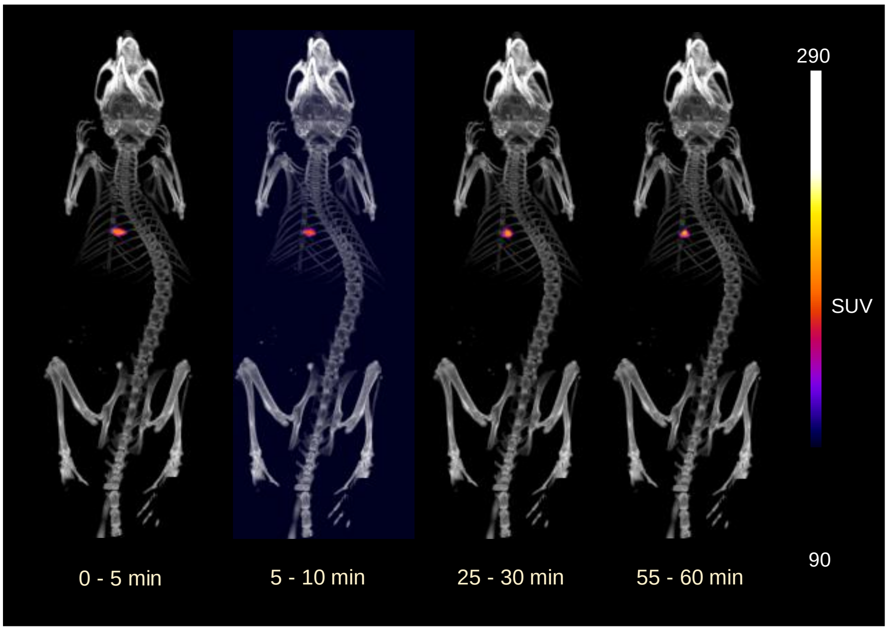

Supplementary Fig. 11│*In vivo* PET/CT imaging of a single ^{68}Ga-smSiP-PEG$_{5k}$. *In vivo* PET/CT imaging of a BALB/c mouse at 0 - 5, 5 - 10 and 25 - 30 min after the *i.v.* injection of 0.95 kBq of ^{68}Ga-smSiP-PEG$_{5k}$.

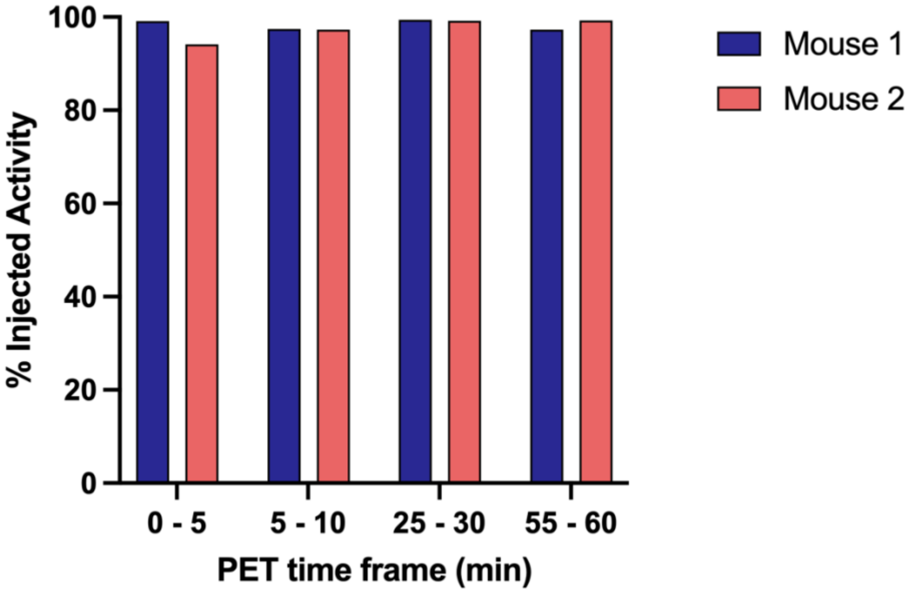

Supplementary Fig. 12│Quantification of PET signal for a single ^{68}Ga-smSiP-PEG$_{5k}$. Quantification of the PET signal represented as % of injected activity for each time frame of the PET reconstruction for mouse 1 injected with 0.95 kBq of ^{68}Ga-smSiP-PEG$_{5k}$ and mouse 2 injected with 2.9 kBq of ^{68}Ga-smSiP-PEG$_{5k}$.

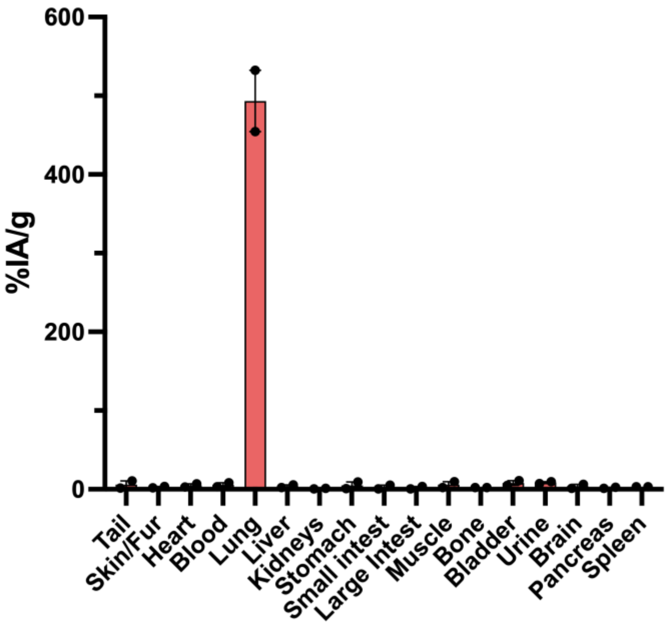

Supplementary Fig. 13│Biodistribution of ^{68}Ga-smSiP-PEG$_{5k}$. Gamma counter biodistribution represented as % IA/g (injected activity per g of tissue) 120 min after *i.v.* injection of a single 0.95 – 2.9 kBq ^{68}Ga-smSiP-PEG$_{5k}$ (n = 2) showing radioactive signal only in the lungs. Data are presented as mean ± SD.

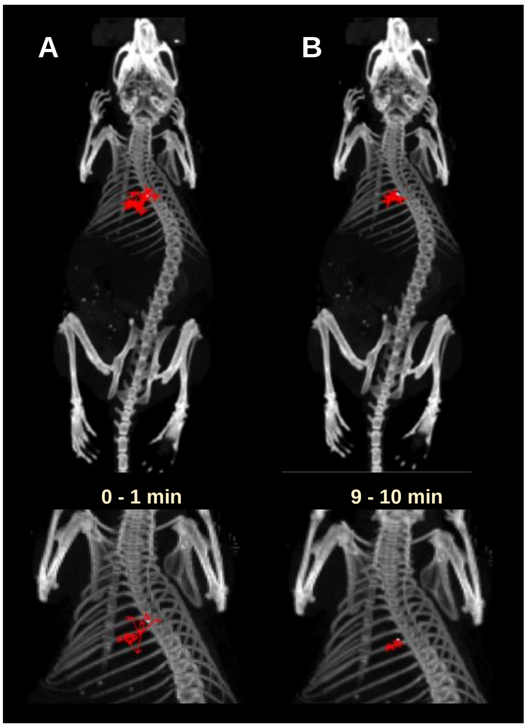

Supplementary Fig. 14│PEPT images for ^{68}Ga-smSiP-PEG$_{5k}$. PEPT technique applied to a Balb/c mouse using the listmode data generated from the PET acquisition after the injection of 0.42 kBq of ^{68}Ga-smSiP-PEG$_{5k}$. Red arrows depict the real-time trajectory of the particle for whole-body and magnified image of coronal MIP at: **A.** 0 – 1 min and **B.** 9 – 10 min after particle injection.

Supplementary Table 1│Links to PEPT videos. Online links to videos generated from the PEPT data for all mice and particles (^{68}Ga-smSiP and ^{68}Ga-smSiP-PEG$_{5k}$).

	Scale	Timeframe	filename	Online link
**^{68}Ga-smSiP (mouse 1) With ROIs**	Whole body	0 – 60 s	M1_60s	https://figshare.com/s/299978d1f2b5d0e91bb9
	Zoom	0 – 60 s	M1_60 s_zoom	https://figshare.com/s/5a19a10e39d6876caa7c
	Whole Body	3 – 10 min	M1_3-10min	https://figshare.com/s/55546490c1ff0a57b274
	Zoom	3 – 10 min	M1_3-10min_zoom	https://figshare.com/s/6f03b64a12a9c8410281
**^{68}Ga-smSiP (mouse 2) With ROIs**	Whole Body	0 – 60 s	M2_60s	https://figshare.com/s/ce70f3e8ec38a6e89b0c
	Zoom	0 – 60 s	M2_60 s_zoom	https://figshare.com/s/33b1282141933ed7a0b9
**^{68}Ga-smSiP (mouse 3)**	Whole body	0 – 60 s	M3_60s	https://figshare.com/s/e55764628118bb798895
	Zoom	0 – 60 s	M3_60 s_zoom	https://figshare.com/s/3331d4a034916842ee0d
**^{68}Ga-smSiP (mouse 4)**	Whole body	0 – 60 s	M4_60s	https://figshare.com/s/8c12cfa72956a5218f52
	Zoom	0 – 60 s	M4_60s_zoom	https://figshare.com/s/c382924eb362e3c063f5
**^{68}Ga-smSiP-PEG$_{5k}$ (mouse 1) With ROIs**	Whole body	0 – 60 s	M1_PEG_60 s	https://figshare.com/s/01d82f5bf4ade8a4f7c6
	Zoom	0 – 60 s	M1_PEG_60s_zoom	https://figshare.com/s/0f8c4b37895df3a3d7e5
	Whole body	8 – 15 min	M1_PEG_8-15mins	https://figshare.com/s/2ffc81a3b00528cac1f0
	Zoom	8 – 15 min	M1_PEG_8-15mins_zoom	https://figshare.com/s/8ab4888d9a3168a7d7cc
**^{68}Ga-smSiP-PEG$_{5k}$ (mouse 2)**	Whole body	0 – 60 s	M2_PEG_60 s	https://figshare.com/s/97dbbd12ed744df728ea
	Zoom	0 – 60 s	M2_PEG_60 s_zoom	https://figshare.com/s/eb8768d11addbcc86b7a
